# Annotations

**Published:** 1893-12-09

**Authors:** 


### ANNOTATIONS.

### The Death of Professor Tyndall.

Professor Tyndall is dead. His was a noteworthy personality, though the *Times* decides that he was "not great perhaps, but considerable." Most brainworkers will sympathise with the sleeplessness which harassed his later years, and which in the end shortened his life. Tyndall impressed the world by means of two striking features. He was an Agnostic, and he was a man of science who believed that the science which he had found good for himself would be good also for the mass of mankind. He did his utmost to enrich all men out of the stores of his own ample knowledge. Concerning Tyndall's Agnosticism, it is perhaps sufficient here to say that his master, Faraday, who was not merely "considerable," but admittedly "great," was as conspicuous for "belief" as Tyndall was for "doubt." On the other feature of his character, that of a generous desire to share his knowledge with the world and to raise the world, if possible, to his own level, it is very pleasant to dwell. He was one of the first to give the now famous Christmas lectures to children at the Royal Institution. The titles, and in many cases the contents of some some of his popular but entirely scientific works are as familiar to the ordinary intelligent reader as household words: for example, "Forms of Water," "Sound," "Heat as a Mode of Motion," "Fragments of Science," "Faraday as a Discoverer," &c. This feature of his character constitutes at least one claim to true greatness. Tyndall was one of the "dauntless three," as they have been called, who fought and won the battle of science in the modern world. Darwin and Huxley were the other two. In our judgment, *pace* the *Times*, posterity will pronounce all three "great," though not all equally great. This great man had his weaknesses—as who has not?—and his contemporaries have probably been a little too conscious of those weaknesses. We have all been too conscious of the weaknesses of one of the great scientist's nearest friends, Carlyle, thanks to the unsympathetic and undiscriminating candour of Mr. Froude; and we are beginning to be ashamed of our pharisaical promptitude in the art of condemnation. Let us not make the same mistake with Tyndall. The fame of Tyndall will survive his peculiarities, and his name will probably prove to be not unworthy to stand side by side with the name of that other Tindale, whose noble boast it was that he would "place an open Bible within reach of every ploughman in England."

### Lord Salisbury's Despondency.

The Marquis of Salisbury, has been described as the victim of "despondency," but the despondency which holds the great statesman by the throat is the natural companion of his time of life. The Marquis is exactly sixty-three years of age. The leading journal may be right as to the fact of the despondency of its illustrious single instance, but we are very unwilling to believe that despondency is the natural companion of all, or even the majority, of men who reach the age of sixty-three. Milton was probably not more than thirty-three when he wrote, "Hence, loathed melancholy, of Erebus and blackest midnight born." Mr. Gladstone is eighty-three, and so far from being the victim of despondency, he seems to be quite the jauntiest young octogenarian of his generation. Physiology offers a better explanation of despondency than the age of sixty-three. The Marquis of Salisbury is a student—a student and not an active man. He works too much with his brain and too little with his body. He has probably always done this since his boyhood, and at any rate for the last thirty years. As the result of thirty years of unredressed physiological balance, the adipose tissue has accumulated at the expense of the muscular. The brain, having been long accustomed to exact more than its fair share of nutriment, finds itself, now that the energy of the cardiac muscle is impaired, compelled to do with less than its fair share. Hence its melancholy and tendency to despondency. It is possible that the great Marquis, by invincible resolution, might yet bring about a brighter and happier condition of things for himself. But, at any rate, so conspicuous an object lesson should not be lost upon younger students and intellectual workers. Sixty-three should be a bright, happy, and victorious period of life. It is so in the average man who has maintained a just balance between muscle and brain activity; but not in others. Robust exercise, abundance of fresh air, and abundance of intellectual work are the conditions of health and happiness for the student. Brain work, without adequate physical exercise and pure air, inevitably leads to despondency at sixty-three.

### A Medical Lovers' Quarrel.

When a young and ardent lover wooes a lady several years his senior he usually finds the conquest comparatively easy. This rule appeared to be in the way of receiving an added confirmation in the case of the London and Counties Medical Protection Society, which recently made overtures of marriage to the Medical Defence Union. For a time "all went merry as a marriage bell." The matchmakers on both sides were confident of a speedy and happy union. But all at once we hear of collapse, quarrels, and a general breakdown. These two grave and responsible societies have withdrawn from each other to the utmost limits of their respective tethers. We are not in a position to apportion the blame, but inasmuch as the union seemed to be eminently suitable and desirable we cannot but feel that there has been a lack of diplomatic tact and talent somewhere. Did the older organisation, priding itself upon its age, demand too much? And did the younger, full of confidence in its youth and rapid success, assume too easy a conquest? It is whispered that the younger society asked for amalgamation, but that the older responded by offering absorption: absorption, and nothing but absorption. The Medical Defence Union was willing to eat up the Protection Society, but not to marry it. Nay, even the eating up was conditioned by the willingness or otherwise of the Protection Society to make itself easy of deglutition by breaking off one or two knobs in the shape of members of council disapproved by the Defence Union. This, we believe, was more than the Protection Society could stand. It loyally refused to heave its supposed Jonah or Jonahs overboard. Hence the suspension of the negotiations. But we submit that politicians of a more practical character would not have been baulked of a great project of union by such very small difficulties. Clearly medical men are wanting in the capacity for affairs.

